# Mapping synaptic glutamate transporter dysfunction *in vivo* to regions surrounding Aβ plaques by iGluSnFR two-photon imaging

**DOI:** 10.1038/ncomms13441

**Published:** 2016-11-11

**Authors:** J. K. Hefendehl, J. LeDue, R. W. Y. Ko, J. Mahler, T. H. Murphy, B. A. MacVicar

**Affiliations:** 1 Djavad Mowafaghian Centre for Brain Health, Faculty of Medicine, University of British Columbia, 2211 Wesbrook Mall, Vancouver, British Columbia, Canada V6T 1Z3; 2 Hertie-Institut für klinische Hirnforschung, Otfried-Müller-Strasse 27, 72076 Tübingen, Germany

## Abstract

Amyloid-β (Aβ) plaques, a hallmark of Alzheimer's disease (AD), are surrounded by regions of neuronal and glial hyperactivity. We use *in vivo* two-photon and wide-field imaging of the glutamate sensor iGluSnFR to determine whether pathological changes in glutamate dynamics in the immediate vicinity of Aβ deposits in APPPS1 transgenic mice could alter neuronal activity in this microenvironment. In regions close to Aβ plaques chronic states of high spontaneous glutamate fluctuations are observed and the timing of glutamate responses evoked by sensory stimulation exhibit slower decay rates in two cortical brain areas. GLT-1 expression is reduced around Aβ plaques and upregulation of GLT-1 expression and activity by ceftriaxone partially restores glutamate dynamics to values in control regions. We conclude that the toxic microenvironment surrounding Aβ plaques results, at least partially, from enhanced glutamate levels and that pharmacologically increasing GLT-1 expression and activity may be a new target for early therapeutic intervention.

## Abstract (web summary)

In Alzheimer's disease (AD), neural hyperactivity has been shown to occur in the regions surrounding Aβ plaques. Here, the authors use *in vivo* two-photon imaging in mouse models of AD and report abnormal glutamate dynamics in the vicinity of plaques which can be partially restored via GLT-1 upregulation through Ceftriaxone treatment.

## Graphical abstract

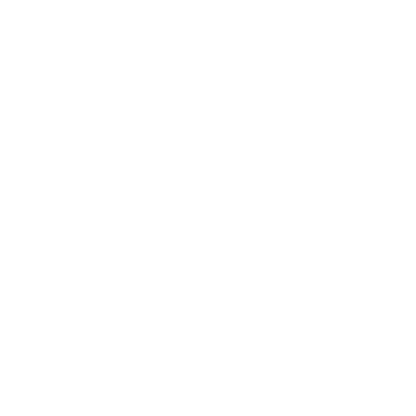

Alzheimer's disease (AD) is a progressive neurodegenerative disorder and, with no cure available, it imposes a major burden on society. The pathological hallmarks of AD include the extracellular accumulation of amyloid plaques and intracellular protein inclusions known as neurofibrillary tangles^1^,^2^,^3^,^4^. Positron emission tomography imaging using Pittsburgh compound B has shown that cerebral Aβ plaque deposition is an early and predictive marker for the progression of preclinical to symptomatic AD^5^,^6^. Recent publications have revealed many interesting aspects of cell dysfunction in relation to Aβ deposits^7^,^8^,^9^,^10^,^11^,^12^. A current working hypothesis is that soluble Aβ adheres and combines with resident Aβ plaques leading to progressive plaque growth^13^,^14^. Although plaques consist of insoluble Aβ, each plaque may create a toxic microenvironment because soluble species continuously bind and unbind from its surface^7^,^8^. The toxic microenvironment hypothesis is supported by observations of neuronal dysfunction and decreased spine density in the immediate regions surrounding Aβ plaques^15^,^16^. Furthermore, hyperactive neurons, microglia and astrocytes with elevated intracellular calcium levels have been reported in the close proximity of Aβ plaques^9^,^10^,^12^,^17^,^18^. A shortcoming of the amyloid cascade hypothesis is that there are no clear connections between the onset of plaque deposition and the progression of cognitive impairment and neuronal loss^19^,^20^. In contrast, there are stronger correlations with the levels of soluble Aβ species^21^,^22^,^23^ and cognitive impairment as well as neuronal loss.

In this study we investigated whether changes in glutamate dynamics in the microenvironment surrounding Aβ deposits could contribute to neuronal dysfunction preceding the onset of neuronal death. This may establish an early link between Aβ toxicity and neuronal dysfunction. A genetically encoded fluorescent glutamate indicator, iGluSnFR, was used to detect the presence and time course of glutamate dynamics *in vivo* with two-photon and wide-field fluorescence microscopy^24^. It allows for unprecedented spatial and temporal resolution of local glutamate concentration on a subsecond timescale. Rapid glutamate uptake from the synaptic cleft, principally by the astrocyte glutamate transporter excitatory amino-acid transporter 2 (EAAT2 in humans or the homologue glutamate transporter 1 (GLT-1) in mice)^25^ permits precise and fast synaptic transmission. Impairment of glutamate clearance in the extracellular space in AD^26^,^27^,^28^ might underlie the reported upregulation of Ca^2+^signalling in neurons and astrocytes in the microenvironment of Aβ deposits and could contribute to excitotoxicity, which eventually leads to cell death. EAAT-2 activity has been reported to be significantly reduced in early stages of AD correlating well with cognitive decline in AD patients^29^. Moreover, studies of a heterozygous GLT-1 knockdown in an AD mouse model showed exacerbated cognitive decline further supporting the theory that dysfunction of the astrocyte glutamate transporter is involved in AD pathogenesis^30^,^31^. Recent *in vitro* results suggest that Aβ species are responsible for the loss of GLT-1 expression as a part of their toxicity to astrocytes^32^. Therefore, we treated APPPS1 mice with ceftriaxone to determine whether GLT-1 expression and/or transporter activity could be upregulated around plaques and whether this would restore normal glutamate dynamics in this region. Our results indicate that ceftriaxone helps to partially restore glutamate dynamics and chronically elevated levels of glutamate. Thus, it reduces the pathological impact of Aβ deposits and surrounding prefibrillary forms of Aβ.

## Results

### Wide-field imaging of glutamate dynamics

**Figure 1:**
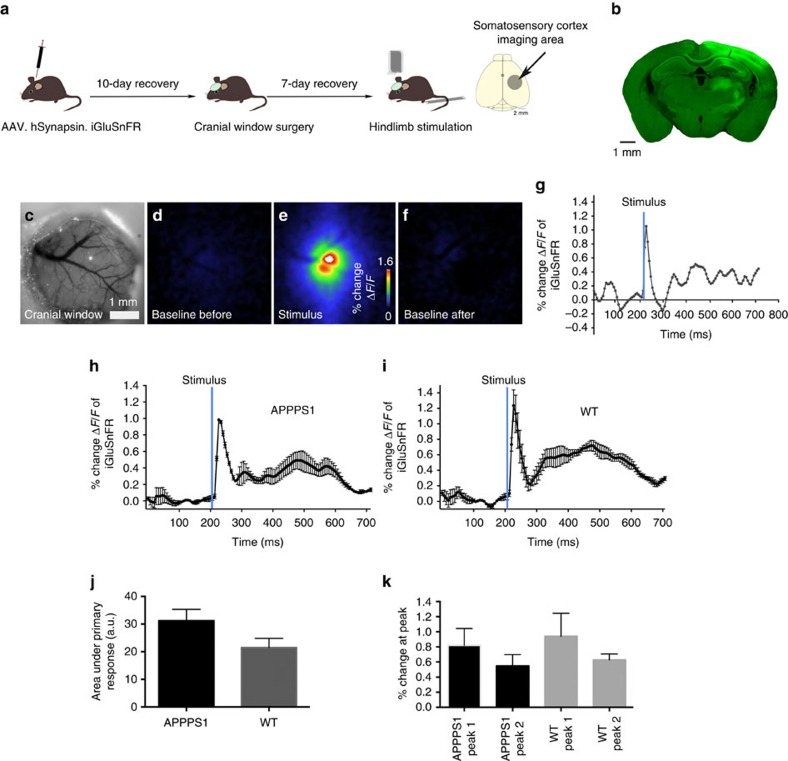
Wide-field imaging of glutamate dynamics do not show significant changes. (**a**) Intracortical injection of AAV.hSynapsin.iGluSnFR in somatosensory cortex area coding for hindlimb (*n*=9 transgene positive animals, 7 age-matched WT animals) followed by a cranial window surgery and mechanical hindlimb stimulation. Animals were head fixed and anaesthetized with isoflurane (1%) during imaging. (**b**) Pattern of iGluSnFR expression in the somatosensory cortex after viral injection. (**c**) The complete cranial window (3.5 mm diameter) was used for the analyses. A stimulus-locked response was detected on hindlimb stimulation (**e**) with baseline levels shown before and after stimulation (**d**,**f**). The respective trace of Δ*F*/*F* also displaying the response on stimulation is shown in **g**. Mean of all recorded traces in APPPS1 (**h**) and WT animals (**i**). (**j**,**k**) However, when analysed at this scale no difference of the measured area under the response between APPPS1 and WT animals (*P*=0.09) or the percentage change (*P*=0.56) of the glutamate response could be detected. Error bars indicate s.e.m.

We first ensured that changes in iGluSnFR fluorescence in mouse brains injected with iGluSnFR (Fig. 1a) were due to evoked synaptic release by examining the transient changes in fluorescent intensity evoked by visual or hindlimb stimulation (APPPS1 mice *n*=9; wild-type (WT) mice *n*=9). Figure 1b shows iGluSnFR expression in the hindlimb area of the somatosensory cortex. The complete cranial window (Fig. 1c, ∼3.5 mm diameter) was used to analyse the stimulus-evoked signal. Wide-field imaging of the prestimulus baseline controls followed by the increase in iGluSnFR fluorescence on hindlimb stimulation and subsequent recovery to baseline levels is shown in Fig. 1d–f. The respective trace of the alterations in Δ*F*/*F*_0_ shows a clear increase on stimulation of the hindlimb (Fig. 1g, for video see Supplementary Video 1).

Analysing the overall changes in iGluSnFR fluorescence with wide-field imaging showed no significant differences between the APPPS1 and WT animals, in area under the curve of the primary peak or the maximum peak amplitude of the iGluSnFR signal (Fig. 1h–k). This indicates that, at the mesoscale (Fig. 1a, ∼3.5 mm), glutamate dynamics appear relatively normal although there was a trend to decreased signals in the APPPS1 mice.

A secondary peak was observed in a much wider cortical area than the primary initial peak that was dominant at the centre of the area responding to the stimuli. The subsequent two-photon laser scanning microscopy (tplsm) experiments were all performed in the centre of the area responding to the stimuli to focus on the primary response peak.

### Glutamate dynamics at Aβ plaques in anaesthetized APPPS1

Macroscopic approaches may obscure pathological changes of brain function in regions adjacent to Aβ plaques in AD models. Thus, we next tested our hypothesis that changes in glutamate signalling occur in discrete regions that surround Aβ plaques. To ensure that the detected changes in glutamate were within the centre of the area responding to the stimuli all animals were mapped for the respective region using wide-field imaging before data acquisition in the tplsm. This way we ensured that the primary response peak was most prominent and plaques were chosen accordingly within this area.

**Figure 2:**
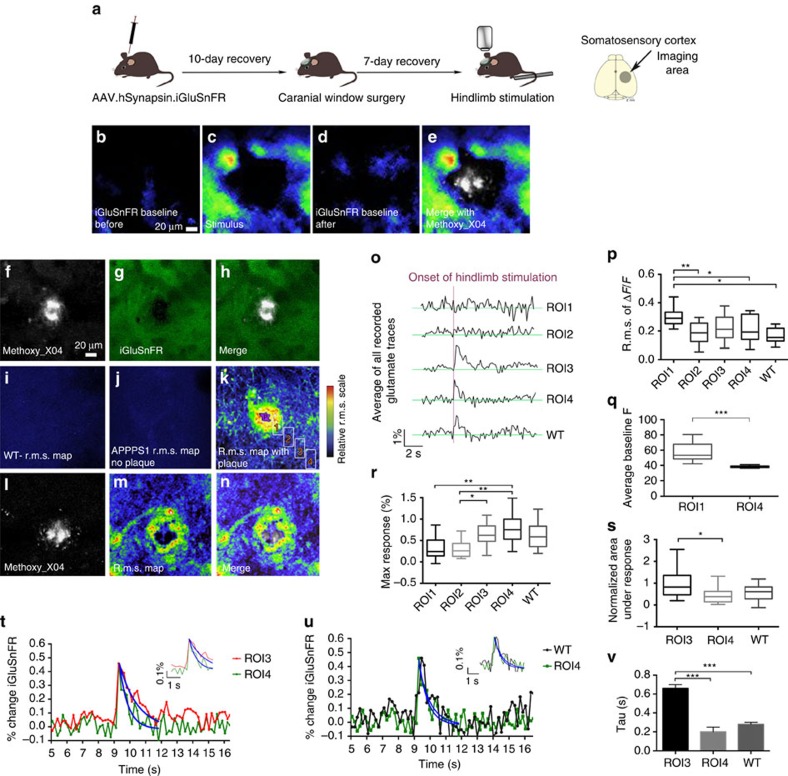
Glutamate dynamics are pathologically altered around Aβ plaques in anaesthetised APPPS1 mice. (**a**) AAV.hSynapsin.iGluSnFR in somatosensory cortex area (APPPS1 mice *n*=9, WT mice *n*=9; 3–7 imaged brain areas per mouse). (**b**) A stimulus-locked response was detected on hindlimb stimulation (**c**) with baseline levels shown before and after stimulation (**b**,**d**). A merge of the stimulus response and a Methoxy_X04-stained amyloid deposit is shown in **e**. (**f**) Methoxy_X04-stained amyloid deposit. (**g**) iGluSnFR expression shown in green around the Aβ plaque shown in **f**. (**h**) Merge of amyloid deposit and iGluSnFR. (**i**–**n**) Glutamate fluctuations were imaged using tplsm and are displayed in r.m.s. maps. WT animals (**i**) and APPPS1 animals that were imaged in areas where no plaques were found (**j**) show a smooth r.m.s. map with no regional differences. (**k**) APPPS1 animals show high regional differences in r.m.s. map with altered glutamatergic activity near plaque. Numbered boxes indicate analysed ROIs with increasing distance to plaque edge. (**l**) Methoxy_X04-stained Aβ plaque. (**m**) The highest average r.m.s. was measured in the vicinity of Aβ plaques. (**n**) Merged image of plaque (**l**) and r.m.s. map (**m**). (**o**) Traces representing glutamate dynamics differ significantly in relation to Aβ plaque distance. (**p**) The average r.m.s. of Δ*F*/*F* is significantly different in ROI1 in comparison with all other ROIs and WT animals. (**q**) Comparison of average baseline fluorescence in ROIs 1 and 4. ROI1 shows a significantly higher level of relative baseline fluorescence in comparison with ROI4. (**r**) The maximal response to the stimulation was detected in the ROI imaged farthest away from the Aβ plaque (ROI4), which was not significantly different from the responses detected in WT animals. (**s**) A significant difference in the area under the response between ROI3 and ROI4 was detected. No difference for the area under the response was found when comparing ROI4 with WT animals. (**t**–**v**) The decay rates of glutamate calculated for ROIs 3 and 4, as well as WT animals are significantly different. (**t**) Normalized glutamate dynamics on stimulation in per cent change is shown to illustrate the significantly different decay rates between ROIs 3 and 4. The rate of decay significantly increased from ROI4 to ROI3 indicating that glutamate is not taken up with the same efficiency when approaching the plaque. (**u**,**v**) Comparing the decay rate of glutamate in ROI4 with the one obtained from WT animals no difference could be detected. Error bars indicate s.e.m. ***P<0.001, **P<0.01, *P<0.05.

Using tplsm the magnitude and timing of glutamate transients were detected at higher spatial resolution (150 × 150 μm) in the regions surrounding amyloid deposits (APPPS1 mice *n*=9, WT mice *n*=9; 3–7 imaged brain areas per mouse; Fig. 2a). As in the case of wide-field imaging we first ensured that changes in iGluSnFR fluorescence during tplsm imaging in mice injected with iGluSnFR (Fig. 2b–e) were due to evoked synaptic release by examining the transient changes in fluorescent intensity evoked by visual or hindlimb stimulation. Furthermore, to ensure that the detected signal is glutamate dependent, we manipulated the glutamate-signalling pathway using two approaches (*n*=2 animals). First, we blocked presynaptic release of glutamate using cadmium chloride (Cd^2+^), which abolished the stimulus-evoked response (Supplementary Fig. 1A,C). Second, threo-beta-benzyloxyaspartate (TBOA) was applied to block the uptake of glutamate, which resulted in prolonged glutamate responses and a higher baseline fluorescence (Supplementary Fig. 1B,D). To exclude a localized expression difference of iGluSnFR in the direct surroundings of amyloid deposits we checked expression levels using pure iGluSnFR fluorescence. Figure 2g,h shows a homogeneous expression of iGluSnFR around the Aβ plaque shown in f.

The characteristics of unsynchronized, local and spontaneous glutamate transients were investigated by quantifying the magnitude of signal fluctuations at each pixel over time and by calculating the root mean square (r.m.s.) of the iGluSnFR signals. Alterations in r.m.s. signals representing glutamate fluctuations in different regions around the plaques were compared and r.m.s. variations are illustrated as colour-coded heat maps (Fig. 2i–k,m,n). R.m.s. heat maps from APPPS1 animals in imaging locations adjacent to plaques show clear regional differences (Fig. 2k,m,n). The highest fluctuations in glutamate dynamics and thus the highest average r.m.s. were measured in the direct vicinity of the plaque (within a distance of 20 μm from the outer edge of the plaque, Fig. 2k,m,n) indicating that the r.m.s. signals are greater the closer the region is to the plaque border. This region also exhibited the highest values within the obtained r.m.s. maps and iGluSnFR fluctuations of Δ*F*/*F*_0_ indicating the highest spontaneous glutamate levels (Fig. 2m,o). In contrast to these local alterations in glutamate dynamics, WT animals display a low and relatively homogeneous average r.m.s. throughout the imaged field of view (Fig. 2i). This indicates that no prolonged presence of extracellular glutamate transients could be detected in WT animals. Interestingly, when spontaneous glutamate fluctuations were imaged over time at locations in APPPS1 mice in which there were no amyloid deposits, a low and homogeneous average r.m.s. was measured, similar to that observed in WT animals (Fig. 2j). Therefore, the largest dynamic changes in spontaneous glutamate concentrations are locally restricted to areas adjacent to amyloid deposits.

Glutamate transients evoked by sensory stimulation also showed marked differences in the regions close to amyloid deposits. Hindlimb stimulation evoked a transient glutamate signal from synaptic release that had similar characteristics in somatosensory cortex in WT mice and in APPPS1 mice in regions distant from plaques (compare ROI4 with WT in Fig. 2o,u). The secondary lower amplitude glutamate peak observed broadly in the wide-field imaging was not usually apparent in two-photon imaging around plaques. The source of this difference is unknown but might be caused by imaging different volumes of tissue as mentioned in the Methods section. However, to ensure that the differences between wide-field imaging and tplsm were not due to the difference in scan speed we selected a smaller region of 16 × 128 pixels to reach a scan speed of 57 Hz. We found no differences in the rate of glutamate decay, r.m.s., area under the response or maximum response in comparison with the slower scan speed of 5.92 Hz (Supplementary Fig. 2A–F). Furthermore, the response curves also only show one primary response peak.

Discrete regions of interest (ROI) at increasing distances from the boundary of methoxy_X04-stained Aβ plaques were analysed separately to characterize the response alterations with respect to the distance from the plaque. The greatest fluctuations in spontaneous glutamate transients quantified as r.m.s. were observed in ROI1 in comparison with all other ROIs (Fig. 2p). However, hindlimb stimulation did not evoke glutamate transients in ROI1 or 2, whereas time-locked transients were observed in ROI3 and ROI4 (Fig. 2o,r,t). To exclude the possibility that a stimulus-evoked response of glutamate is hidden in the high fluctuation in ROI1, the hindlimb stimulation was repeated using 100 trials. The average r.m.s. of Δ*F*/*F* was still significantly higher in ROI1 in comparison with all other ROIs and the mean of all measured glutamate traces still did not result in a stimulus-locked response in ROIs 1 and 2 (Supplementary Fig. 3). This suggests that the high degree of spontaneous glutamate dynamics measured as r.m.s. signals in ROI1 was likely due to chronic but spontaneous elevations in glutamate within this area. To test this hypothesis, we measured the baseline fluorescence of iGluSnFR in ROI1 and 4. ROI1 showed significantly higher baseline fluorescence in comparison with ROI4 supporting the hypothesis that the detected fluctuations are the result of chronically higher levels of glutamate (Fig. 2q). The first stimulus-locked response was detected in ROI3 that is located 40–60 μm from the edge of a given amyloid deposit. The maximum stimulation-evoked glutamate response in ROI3 was lower and the area under the response was larger than in ROI4 (Fig. 2r,s). In contrast, ROI4 did not show any significant differences in these parameters in comparison with WT animals (*τ*=0.28 s, s.e.m.=0.02; Fig. 2r,s). The characteristic decay time constant (tau) of the stimulation-evoked glutamate response decay in ROI3 (*τ*=0.66 s, s.e.m.=0.04) was significantly slower from the response obtained in ROI4 (*τ*=0.23 s, s.e.m.=0.02; Fig. 2t). The decay time constant in ROI4 (80–100 μm plaque distance) did not differ from that of WT (Fig. 2u). These results indicate that the characteristics of the sensory stimulation-evoked glutamate transients progressively changed closer to the plaque until evoked signals were lost in ROI1 and 2. The increased decay time constant (tau) of the iGluSnFR signal in ROI3 versus the more distal ROI4 (Fig. 2t,v) suggested that glutamate transients were prolonged and clearance reduced within this region. These results clearly demonstrate that the characteristics of glutamate dynamics varied depending on distance from the plaque.

It is important to note that even in the negative animals the iGluSnFR response to stimulation is not uniform throughout the field of view even in the region of the peak response. Thus, to ensure that the observed local differences in the pattern of the responses was not obtained simply by chance due to the non-uniform nature of the iGluSnFR signal we randomly generated a position to serve as the plaque location and placed the four ROIs at the correct distance from the ‘plaque' location on transgene negative animal recordings (also chosen at random from amongst our data set). This process was repeated for 500 artificial plaque locations and the ROI responses were then averaged (Supplementary Fig. 4D, *n*=9 animals). We measured the average responses in all ROIs and found no difference between the maximum response amplitudes or the decay constants between ROIs (Supplementary Fig. 4B,C). This analysis demonstrates that plaques are indeed surrounded by ROIs with altered response amplitudes and decay times in APPPS1 animals.

The observed spontaneous and stimulus-evoked fluctuations of extracellular glutamate might also cause fluctuations of intracellular calcium concentrations in neurons in close proximity to amyloid deposits. To test this hypothesis, we combined glutamate and calcium recordings by co-expressing iGluSnFR with the red calcium indicator jRGECO. Co-excitation of the two reporters was possible using an excitation wavelength of 950 nm. iGluSnFR responses to hindlimb stimulation remained similar in the combined experiment and are shown in Supplementary Fig. 5D (*n*=3 animals, 3–4 imaged brain regions). Again no stimulus-locked iGluSnFR responses were observed in ROI1 or ROI2. The return to baseline of the signal in ROI3 is notably longer than ROI4. Calcium transients recorded in the same field of view simultaneously with the iGluSnFR signal showed responding cells (somas) in all regions (Supplementary Fig. 5C). Such sensory-evoked calcium signals have been reported by others^7^. However, it should be noted that the lowest proportion of responding cells was observed in ROI1 and that the fraction of responding somas increased with increasing distance from the plaque. We counted the numbers of responding versus non-responding cells and grouped them into four groups corresponding to the distances of ROIs 1–4 based on the distance between the plaque and the soma. This is shown in Supplementary Fig. 5E. In ROI1 ∼20% of cells responded compared with ∼65% in ROI4.

### Glutamate dynamics around Aβ plaques in awake APPPS1

We examined the characteristics of glutamate release evoked by sensory stimulation in awake animals to ensure that alterations in glutamate uptake around amyloid deposits were not due to anaesthesia (APPPS1 mice *n*=7, WT mice=7; 3–7 brain areas per mouse). We examined the visual cortex in awake animals, as this would avoid interfering signals from constant limb movement, which would obscure somatosensory stimulation-evoked changes. Tplsm of iGluSnFR was repeated in the primary visual cortex of awake state APPPS1 and WT controls. Light stimulation was used to evoke glutamate transients in the visual cortex and the same analysis that was done in the somatosensory cortex was repeated. Similar disruptions in glutamate dynamics were observed close to Aβ plaques even though larger maximum responses to stimulation and higher r.m.s. values of Δ*F*/*F*_0_ were observed in awake animals compared with anaesthetized animals.

**Figure 3:**
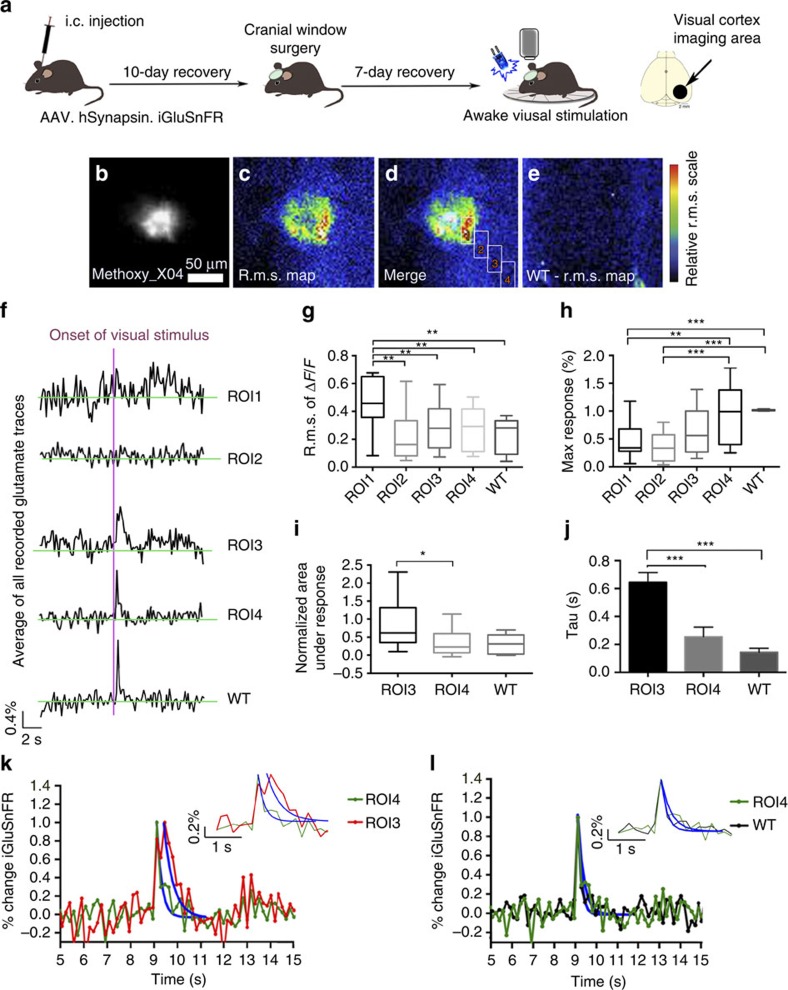
Glutamate dynamics are altered due to Aβ plaque presence in awake APPPS1 mice. (**a**) Intracortical injection (i.c.) of AAV.hSynapsin.iGluSnFR in primary visual cortex area (APPPS1 mice *n*=7, WT mice=7; 3–7 imaged brain areas per mouse). (**b**) Methoxy_X04-stained amyloid deposit. Scale bar, 50 μm. (**c**) APPPS1 animals show significant alterations in glutamatergic activity near plaque. (**d**) Merge of amyloid deposit and r.m.s. map of plaque shown in **b** with boxes indicating analysed ROIs 1–4. (**e**) WT animals display a lower average r.m.s. with no region-specific differences. (**f**) Traces representing glutamate dynamics differ significantly in relation to the distance from Aβ plaques. (**g**) The average r.m.s. of Δ*F*/*F* is significantly different in ROI1 in comparison with all other ROIs and WT animals. (**h**) The maximal response to the stimulation was detected in the ROI imaged farthest away from the Aβ plaque (ROI4), which was not significantly different from the responses detected in the WT animals. (**i**) After normalization a significant difference in the area under the response between ROI3 and ROI4 was detected. No difference was found when comparing ROI4 with WT animals. (**j**) The decay rates of glutamate calculated for ROIs 3 and 4, and WT animals are significantly different. (**k**) Normalized glutamate dynamics on stimulation in percent change is shown to illustrate the significantly different rates of decay between ROIs 3 and 4. The rate of decay significantly increased from ROI4 to ROI3 indicating that glutamate is not taken up with the same efficiency when approaching the plaque. (**l**) Comparing the decay rate of glutamate in ROI4 to the one obtained from WT animals no difference could be detected. Error bars indicate s.e.m. ***P<0.001, **P<0.01, *P<0.05.

R.m.s. maps of iGluSnFR signals in awake APPPS1 animals exhibited large regional differences in their average intensity (Fig. 3c,d) with again the largest fluctuations observed in ROI1 (Fig. 3g). In WT animals the average r.m.s. map was relatively homogeneous throughout the field of view (Fig. 3e). As observed during the hindlimb stimulation experiments in the anaesthetized animals, there were also qualitative differences in the stimulus-locked response in awake mice from visual stimulation at increasing distances from a given amyloid deposit (Fig. 3f–j, ROIs 1–4). The maximum response (Fig. 3h) and the smallest area under curve of the stimulation-evoked response (Fig. 3i) were again measured in ROI4 and were not significantly different to data from WT animals. ROI3 displayed a smaller maximum response and a significantly larger area under the peak response in comparison with ROI4 or WT animals (Fig. 3h,i). In addition, changes in decay constant were observed such that in ROI3 (*τ*=0.64 s, s.e.m.=0.07) there was a significantly longer decay constant of the iGluSnFR signal in comparison with ROI4 (*τ*=0.25 s, s.e.m.=0.07) or WT animals (*τ*=0.14 s, s.e.m.=0.03; Fig. 3j–l). The decay rate in ROI4 (80–100 μm plaque distance) did not differ from the one in WT animals (Fig. 3l).

### Chronic elevation of glutamate fluctuation around Aβ plaques

**Figure 4:**
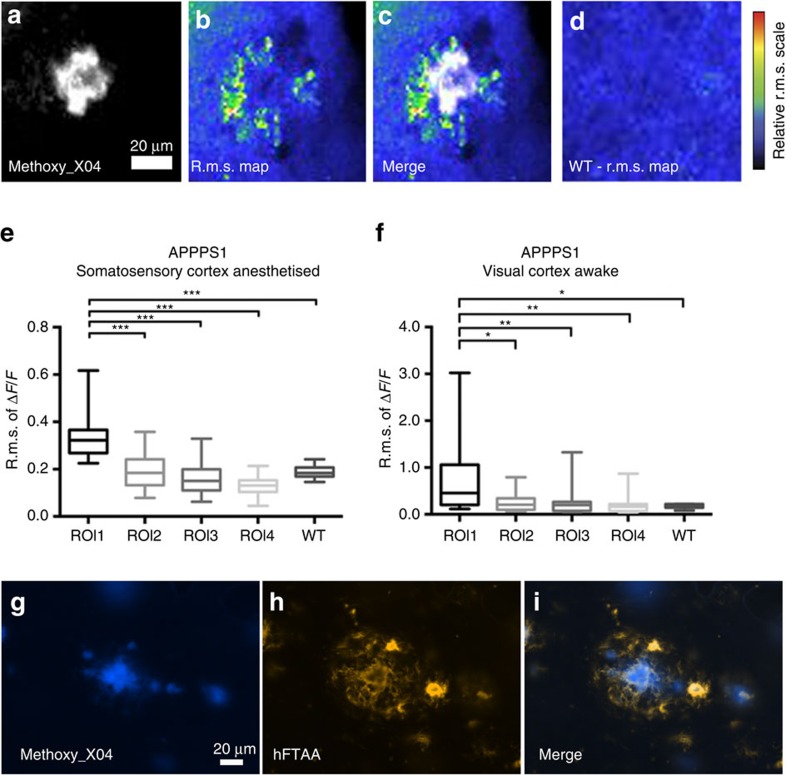
Spontaneous recordings show chronically changed glutamate dynamics. Intracortical injection of AAV1.Syn.Flex.NES-jRGECO1a.WPRE.SV40 in somatosensory or visual cortex area (for visual cortex: APPPS1 mice *n*=7, WT mice=7, 3–7 imaged brain areas per mouse; for somatosensory cortex: APPPS1 mice *n*=9, WT mice *n*=9, 3–7 imaged brain areas per mouse) followed by a cranial window surgery and mechanical hindlimb or visual stimulation. Animals were head fixed and anaesthetized with isoflurane (1%) during imaging. Methoxy_X04 was injected 24 h before imaging intraperitoneally to visualize amyloid deposits at an excitation wavelength of 800 nm. Scale bar, 20 μm. (**a**) Methoxy_X04-stained Aβ plaque. (**b**) The average r.m.s. of the magnitude of spontaneous glutamate dynamics over 2.82 min period (Δ*F*/*F*) is shown in false colours and represents the entire imaging period. (**c**) Merge of methoxy_X04-stained amyloid deposit and respective r.m.s. map. (**d**) WT animals display a lower average r.m.s. with no region-specific differences. (**e**,**f**) In both anaesthetized and awake trials in the two cortical areas the r.m.s. of Δ*F*/*F* in spontaneous recordings of all ROIs is still significantly different in ROI1 in comparison with all other ROIs and WT animals. This suggests that the glutamate dynamics are chronically altered in the microenvironment of the plaque and is not purely dependent on a stimulus. (**g**–**i**) Immunohistological staining of APPPS1 mice (*n*=4) showing a methoxy_X04-stained amyloid plaque (**g**) and prefibrillar forms of Aβ in a 20 μm radius around the plaque stained by hFTAA (**h**). (**i**) The merged image shows the conformational differences of the detected Aβ species by methoxy_X04 (mature amyloid) and hFTAA (prefibrillar and mature forms of Aβ). ***P<0.001, **P<0.01, *P<0.05.

As no stimulus-locked response was measured in ROI1 but instead a high average r.m.s. was consistently observed in these experiments in ROI1, we repeated the measurements of spontaneous glutamate fluctuations in the somatosensory and visual cortex regions in the same set of animals to determine if r.m.s. maps during spontaneous recordings displayed higher fluctuations around amyloid deposits in ROI1 (Fig. 4b,c) in the same APPPS1 animals (for visual cortex: APPPS1 mice *n*=7, WT mice=7, 3–7 brain areas per mouse; for somatosensory cortex: APPPS1 mice *n*=9, WT mice *n*=9, 3–7 brain areas per mouse). WT animals again displayed a relatively homogeneous average r.m.s. throughout the imaged field of view (Fig. 4d). In both cortical areas analysed, the average r.m.s. of Δ*F*/*F*_0_ of ROI1 was higher in comparison with all other regions and WT animals (Fig. 2e,f). Owing to the higher average baseline fluorescence of glutamate that was found (Fig. 2q) we conclude that a constantly locally higher level of glutamate fluctuation was detected. This chronically altered state of spontaneous glutamate transients appears to depend on the proximity of amyloid deposits.

To further investigate changes in the microenvironment of amyloid plaques, we examined whether prefibrillary forms of Aβ or higher orders of oligomeric Aβ are found in the immediate regions adjacent to but not part of the actual plaques potentially causing the chronic changes. A novel group of luminescent conjugated poly- and oligothiophenes have been used to stain and detect conformational differences in amyloid structure^33^. Most interestingly, the heptameric oligo-thiophene hFTAA (hepta-formylthiophene acetic acid) stains both mature amyloid fibrils and early prefibrillar states of Aβ. We immunohistochemically stained fixed brains (APPPS1 *n*=4 animals) with methoxy_X04, which solely binds to mature amyloid fibrils, in combination with hFTAA. Figure 4g shows the mature amyloid fibrils detected by methoxy_X04. Figure 4h shows a different and more diffuse staining pattern extending beyond the Aβ plaque when using hFTAA. The merged image (Fig. 4i) clearly shows a halo of prefibrillar amyloid in the microenvironment of the Aβ plaque (∼20–30 μm from the plaque edge). Thus, it is possible that the hFTAA-stained halo around plaques may indicate that non-mature forms of Aβ cause the pathological and chronic changes in ROI1 in brain tissue adjacent to Aβ plaques. The presence of prefibrillar amyloid might alter the interstitial space and thus change the diffusion of molecules such as neurotransmitters within this area. Even though it is beyond the scope of our report to account for all possible alterations within the interstitial space we tested the rate of Alexa 594 diffusion within the Aβ plaque environment by injecting the dye *in vivo* (Supplementary Fig. 6). The injection and decay of the dye around the plaque is shown in a sequence of three time points (Supplementary Fig. 6C). The following taus were calculated for the decay rate of Alexa 594 in ROI1 tau=11.290±0.2 s and ROI4 tau=11.30±0.2 s. Thus, no significant difference in the rate of dye diffusion as measured by the decay constant of its fluorescence time course was detected in this experiment.

### Reduced GLT-1 expression around Aβ plaques in APPPS1

**Figure 5:**
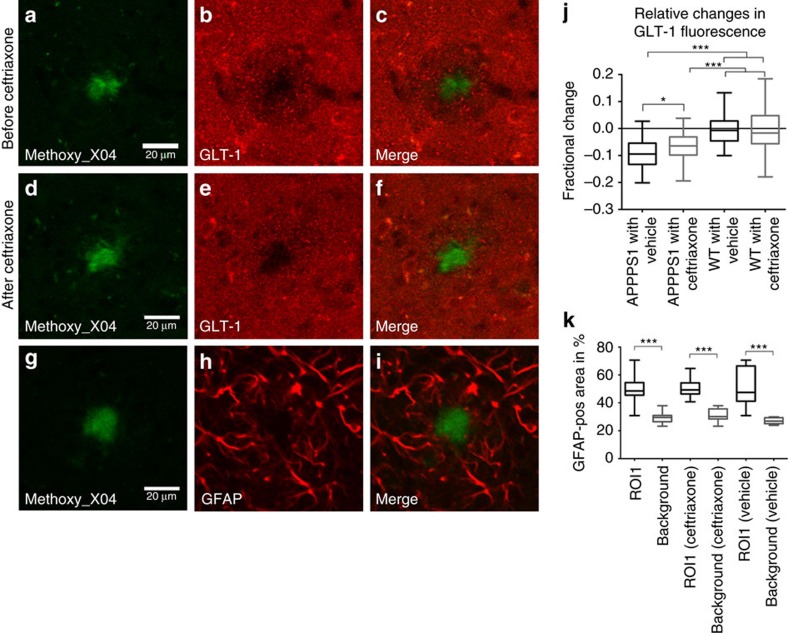
Decrease of GLT-1 around Aβ plaques. Treatment with ceftriaxone partially restores expression levels. Immunohistological staining of GLT-1 before and after ceftriaxone treatment (APPPS1 mice *n*=5, WT mice *n*=4). (**a**) Methoxy_X04-stained amyloid deposit. (**b**) GLT-1 staining of area surrounding Aβ plaque shown in **a** displays a local downregulation of the transporter in a 20 μm radius. (**c**) Merged image of Aβ plaque and GLT-1 staining. (**d**) Methoxy_X04-stained amyloid deposit. (**e**) GLT-1 staining of area surrounding Aβ plaque shown in **d** after treatment with ceftriaxone. Five days of drug treatment partially restored GLT-1 expression in a 20 μm radius around Aβ deposits. (**f**) Merged image of Aβ plaque and GLT-1 staining after ceftriaxone treatment. (**g**) Methoxy_X04-stained amyloid deposit. (**h**) GFAP staining of astrocytes around the Aβ plaque shown in **g**. Downregulation of GLT-1 is thus not caused by the absence of astrocytes in the analysed area. (**i**) Merged image of Aβ plaque and GFAP staining. (**j**) Quantification of relative change in GLT fluorescence after ceftriaxone treatment. (**k**) GFAP-positive pixels were quantified in an annulus extending 20 μm from the edge of the amyloid plaque (*n*=10animals). A similar-sized area that was not plaque associated was used for comparison of GFAP-positive pixels. As the graph shows, ∼50% of the annulus surrounding the plaque is GFAP-positive, while only ∼30% of a plaque-free region is GFAP-positive. The same measurement was performed before and after treatment with ceftriaxone or vehicle. ***P<0.001, *P<0.05.

It was reported that beta-lactam antibiotics, such as ceftriaxone, can significantly and selectively increase the expression and activity of GLT-1 in *in vitro* and *in vivo* studies^34^. Most importantly, the neuro-protective effects of ceftriaxone have been shown in middle cerebral artery occlusion-induced focal brain ischaemia, two-vein occlusion-induced ischaemia and transient forebrain ischaemia models^35^,^36^,^37^,^38^,^39^, and they were thought to be related to the upregulation of GLT-1 (refs 35, 36, 39). Thus, we examined the impact of ceftriaxone in the APPPS1 mouse model. APPPS1 transgenic mice brains were immunohistochemically stained for GLT-1 to determine whether GLT-1 expression was reduced in the tissue surrounding Aβ plaques (APPPS1 mice *n*=5, WT mice *n*=4) visualized in the same sections with methoxy_X04. We observed significant decreases in GLT-1 expression in a 20 μm radius analysed around amyloid deposits (Fig. 5a–c) compared with more distant regions. This distribution suggests that the chronically higher levels of glutamate observed within ROI1 was due to reduced glutamate uptake as a result of lower expression of GLT-1. After ceftriaxone treatment GLT-1 expression in the region adjacent to Aβ plaques was increased and was now similar to GLT-1 expression levels in more distant regions of the brain (Fig. 5d–f,j). Vehicle treatment of APPPS1 animals and treatment of WT animals with ceftriaxone did not result in any significant changes within their groups before and after treatment (Fig. 5j). The reduced GLT-1 expression around Aβ plaques was not due to an absence of astrocytes as immunostaining for glial fibrillary acidic protein (GFAP), and the quantification of GFAP-positive pixels (ROI1 versus background) showed there were no differences within the radius close to the plaques (Fig. 5g–i,k). Treatment with ceftriaxone or vehicle did not result in any differences in GFAP expression pattern (Supplementary Fig. 7).

It is important to note that reports also show that ceftriaxone can alter GLT-1 function without changing overall protein levels^37^,^40^. Thus, even though the upregulation in ROI1 does provide evidence that ceftriaxone has a general effect on GLT-1 in the direct plaque vicinity immunohistological stainings of GLT-1 are not likely to reflect all different stages of GLT-1 from dysfunction to downregulation of the transporter during pathology. Thus, a functional read-out to test possible alterations of GLT-1 unrelated to upregulation of overall protein levels after ceftriaxone treatment using iGluSnFR was needed.

### Upregulation of GLT-1 reduces changes in glutamate dynamics

**Figure 6:**
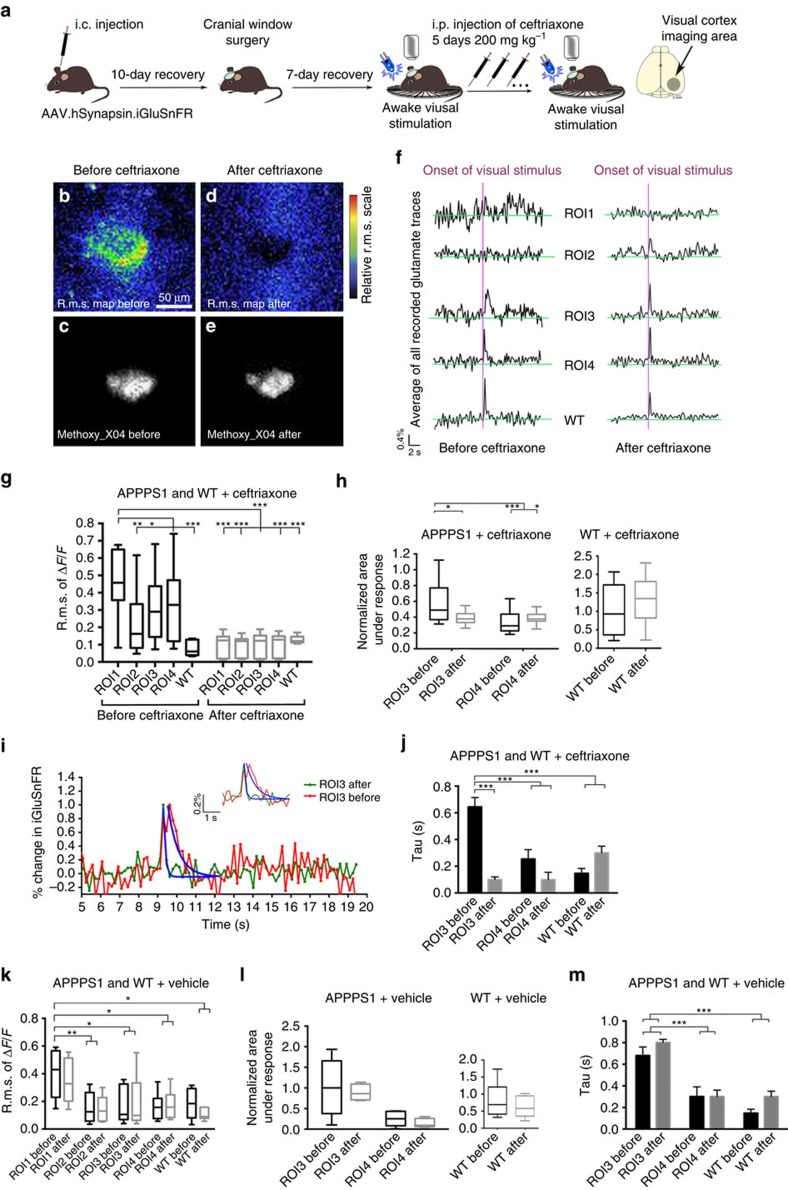
Ceftriaxone partially restores glutamate dynamic deficits around Aβ deposits. (**a**) Intracortical injection (i.c.) of AAV.hSynapsin.iGluSnFR in the primary visual cortex area (APPPS1 mice *n*=7, WT mice=7; 3–7 brain areas per mouse) followed by a cranial window surgery and visual stimulation (single flash) with a blue LED light. Visual stimulation and awake imaging was repeated in the same animals and same ROIs after a 5-day application of ceftriaxone (200 mg kg^−1^). (**b**) Glutamate dynamics were imaged using tplsm. The average r.m.s. of the magnitude of glutamate dynamics over a 20 s period (Δ*F*/*F*) is shown in false colours and represents the entire imaging time. R.m.s. map is shown before (**b**) and after ceftriaxone application (**d**). (**c**,**e**) Methoxy_X04 was injected 24 h before imaging intraperitoneally (i.p.) to visualize the same amyloid plaque before (**c**) and after ceftriaxone application at an excitation wavelength of 800 nm (**e**). Scale bar, 50 μm. (**f**) Traces representing glutamate dynamics change after ceftriaxone application. The purple line indicates the onset of a single LED flash used as a stimulus. The high fluctuation of glutamate measured in the direct plaque vicinity (ROI1) was greatly reduced after ceftriaxone treatment. Furthermore, the quality of the stimulus-locked response in ROI3 is altered. (**g**) After ceftriaxone treatment the regional difference in the r.m.s. maps between all ROIs imaged is significantly lowered. The treatment with ceftriaxone in WT animals did not result in a significant difference when comparing the average r.m.s. before and after treatment. (**h**) After ceftriaxone treatment the difference in the area under the peak between ROI3 and ROI4, which was previously detected, is reversed, suggesting that the characteristics of the response change back to WT levels. The treatment with ceftriaxone in WT animals did not result in a significant difference when comparing the normalized area under the response. (**i**) A time line of the overall normalized glutamate dynamics on stimulation in per cent change is shown to illustrate the significant reversal of glutamate decay before and after ceftriaxone treatment in ROI3. (**j**) The decay rate in ROI3 of glutamate could be restored to a level, which is not significantly from the one measured in ROI4 or WT animals after ceftriaxone treatment. (**k**–**m**) Treatment with vehicle for 5 days in APPPS1 and WT animals did not result in any significant differences for r.m.s. of Δ*F*/*F*, area under the response or rate of glutamate decay. Error bars indicate s.e.m. ***P<0.001, **P<0.01, *P<0.05.

The histological data in Fig. 5 showed that ceftriaxone treatment reversed the selective decrease in GLT-1 expression within the 20 μm radius around amyloid deposits. Thus, we investigated whether this apparent increase in GLT-1 around plaques by ceftriaxone treatment could reverse the high r.m.s. values in ROI1 that indicate greater spontaneous glutamate transients. Furthermore, we tested whether ceftriaxone treatment led to changes in GLT-1 function possibly unrelated to overall protein levels, which would rescue the slower time constant of evoked glutamate decay rates that represent glutamate uptake (Fig. 6a).

Our two-photon imaging set-up allows us to reliably reposition the brain to allow repeated imaging over days and weeks from the same precise coordinates as previously published^14^,^41^. Thus, we were able to revisit previously analysed locations in the visual cortex in awake animals (APPPS1 mice *n*=7, WT mice=7; 3–7 brain areas per mouse) after ceftriaxone treatment. The increase in GLT-1 and hence increased scavenging rate of glutamate has the potential to unmask glutamate transients that were hidden in elevated chronic background fluctuations, such as observed in ROI1. This higher rate of scavenging does however not lead to a loss of iGluSnFR responses, thus giving us the opportunity to detect and compare glutamate traces before and after ceftriaxone treatment. The r.m.s. maps obtained before (Fig. 6b) and after (Fig. 6d) treatment show a clear reduction of the average fluctuations measured over the imaging period. Therefore, treatment with ceftriaxone significantly lowered the chronically upregulated glutamate levels that were detected in the close proximity of the amyloid deposit. An example is illustrated confirming the location of the same amyloid plaque shown before (Fig. 6c) and after (Fig. 6e) treatment. iGluSnFR signals for ROIs 1–4 illustrated before and after ceftriaxone treatment (Fig. 6f) show marked changes in their time courses. The r.m.s. of Δ*F*/*F*_0_ was significantly lowered and reached WT levels in all analysed ROIs when compared before and after treatment. WT animals did not show a change in r.m.s. (Fig. 6g). Moreover, the normalized areas under the response curve in ROI3 were significantly reduced after treatment and were then no longer different from the areas measured in ROI4 or WT animals (Fig. 6h). WT animals did not show a change on treatment when comparing their areas under the response. Most interestingly, when comparing the iGluSnFR signal decay constant in ROI3 before (*τ*=0.64 s, s.e.m.=0.07) and after (*τ*=0.10 s, s.e.m.=0.02) treatment a significant reduction was measured (Fig. 6i). This suggests that the increased expression of GLT-1 rescued the pathological changes in glutamate dynamics within this region (Fig. 6g,j). The decay constant in ROI3 after treatment did not show any significant differences to ROI4 (*τ*=0.09 s, s.e.m.=0.05) or to WT (*τ*=0.3 s, s.e.m.=0.05) (Fig. 6j). WT animals that have been treated with ceftriaxone did not show a significant change in their decay constants (Fig. 6j).

To control for possible effects of the ceftriaxone vehicle a separate group of APPPS1 and WT animals (*n*=5 per group) was treated with the vehicle for 5 days. None of the measured parameters resulted in a significant change due to the vehicle treatment (Fig. 6k–m).

## Discussion

Our results provide a clear insight into a synaptic mechanism contributing to disturbances in glutamate signalling in a model of AD. Tplsm imaging of extracellular glutamate dynamics detected using iGluSnFR revealed impaired glutamate uptake from somatosensory or visual evoked responses in regions adjacent to visualized Aβ plaques. These synaptically evoked glutamate transients showed reduced glutamate clearance rates close to amyloid deposits, and chronic states of prolonged and elevated glutamate levels were detected in the direct vicinity of amyloid plaques. The regions adjacent to plaques also showed reduced expression of the major glutamate transporter, GLT-1. Treatment of APPPS1 mice with ceftriaxone restored GLT-1 expression in the plaque microenvironment almost to control levels and the plaque-associated disturbances in glutamate clearance leading to chronically elevated glutamate concentration were significantly reduced. These results indicate that Aβ plaques are correlated to a regional reduction and dysfunction of GLT-1 that causes impaired glutamate clearance rates. Functional changes to glutamate dynamics and thus possibly GLT-1 activity were detected and rescued at a distance of 40–60 μm (ROI3) from the Aβ plaque edge by ceftriaxone treatment. This indicates that mechanisms unrelated to overall protein levels, such as the dysfunction or mislocalization of GLT-1, may also contribute to the observed pathological alterations. The detailed mechanistic insights thus remain to be investigated. Overall, alterations to glutamate signalling could be an important contributor to synaptic disruption and cognitive impairment in early AD.

Glutamate is the major excitatory neurotransmitter in the brain, and the rapid kinetics of release and clearance from the extracellular space are critical for precisely timed synaptic communication. The principal pathway for glutamate clearance is uptake via the GLT-1 pathway that is expressed in astrocytes. Uptake by astrocytes is essential for glutamate homeostasis that, when disrupted, leads to high levels of glutamate in the extracellular space causing neuronal excitotoxicity and cell dysfunction^11^. The reduction of EAAT2 (the human homologue of GLT-1) activity in the early stages of AD has been reported to be correlated with the cognitive decline seen in AD patients^29^, and studies of heterozygous GLT-1 knockdown mouse models of AD showed exacerbated cognitive decline^30^,^31^. Thus, we re-visited this potential therapeutic candidate with improved scientific methods providing real-time measurements of extracellular glutamate dynamics.

APPPS1 mice develop amyloid plaques by 2 months of age in the neocortex and Aβ42 levels are at least five times higher than those of Aβ40. Though they are associated with neuronal dystrophy and robust astro-and microgliosis^42^ an overall or plaque-associated loss of neurons could not be shown in APPPS1 mice^43^. However, our findings and those of others suggest that we are looking at an early stage of dysfunction in various cell types in the microenvironment of amyloid deposits in this mouse model rather than a model for neuronal loss. This makes the APPPS1 model a valuable tool to mimic early stages in AD in which detrimental downstream events can still be rescued. We hypothesize that the soluble material, known to be mainly Aβ42 in the case of APPPS1 mice, is present in relatively high concentrations in the microenvironment of amyloid deposits. Our staining results with hFTAA support this hypothesis since prefibrillar forms of Aβ were detected in the microenvironment of Aβ deposits. Thus, it is likely that these known toxic oligomeric species of Aβ contribute to the reported alterations in *in vivo* glutamate dynamics as has previously been shown in acute brain slice experiments^44^. To examine alterations in glutamate dynamics in APPPS1 transgenic mice we assessed region-specific alterations in relation to plaque deposition *in vivo* using anaesthetized and awake animals in wide field and tplsm.

Our data show that glutamate dynamics are changed in regions surrounding plaques, and the degree of change increases the closer the tissue is to the edge of the plaque. The time constant of glutamate clearance rates is slower when approaching amyloid deposits, and chronic states of high, fluctuating glutamate concentrations are reached in the direct vicinity of amyloid plaques. Hence, we investigated whether there was a reduction in GLT-1 expression that could lead to a reduction of glutamate reuptake in this area.

Histological staining of GLT-1 showed a significant reduction in the immediate vicinity of amyloid deposits adding evidence to the imaging data that the chronically higher levels of glutamate within ROI1 are due to a reduction in glutamate uptake. Other mechanisms unrelated to overall protein levels of GLT-1 such as receptor dysfunction were investigated by using iGluSnFR as a functional read-out. The beta-lactam antibiotic, ceftriaxone has been widely used to upregulate GLT-1 transporters up to fivefold and also has been shown to alter GLT-1 function and activity independent of overall protein levels^34^,^37^,^40^,^45^,^46^. We thus aimed at reversing higher extracellular glutamate levels and slower glutamate decay rates by increasing glutamate reuptake via GLT-1 transporters. The treatment of APPPS1 animals with ceftriaxone resulted in an increase in GLT-1 in histological staining in ROI1 and a partial rescue of the observed glutamate alterations measured by tplsm of iGluSnFR. The treatment reduced the prolonged decay rates in ROI3 significantly to levels comparable to those in ROI4 and WT animals. Hence we conclude that glutamate dynamics were restored within this region by the treatment. Even though we could not detect a stimulus-locked response in ROI1, we were able to reduce the high average glutamate fluctuations in the direct plaque vicinity to WT levels. The continued lack of a stimulus-locked response in ROI1 might be caused by neuronal dysfunction, which is already also prevalent within this area^7^. It has been shown that a large number of neurons (up to 50%) in the primary visual cortex can display functional impairment without obvious behavioural deficits in transgenic mice. This hints towards possible compensatory mechanisms that are able to sustain physiological activities even in circuits that contain a large number of dysfunctional neurons^7^. It is thus important to note that when using wide-field imaging to record glutamate dynamics on a more global level no significant differences between APPPS1 and WT animals were found (Fig. 1). This comparison indicates that the changes are very region specific and subtle and will be lost if not analysed with the necessary spatial resolution. Interestingly, Zumkehr *et al*.^32^ conducted behavioural tests in 3xTg-AD mice after ceftriaxone treatment of 2 months and found a significant cognitive improvement in the Morris water maze and novel object recognition tests.

Overall, these findings point towards a contribution of high glutamate concentrations to the reported excitotoxic microenvironment surrounding amyloid deposits. High levels of extracellular glutamate could mediate the reported upregulation of Ca^2+^signalling and overall hyperactivity in the microenvironment of Aβ deposits and could contribute to the progressive synaptic disruption and cell death. Thus, early changes in glutamate dynamics could be used to detect the early development of detrimental excitotoxic downstream effects. However, studies in patients would require spatial and temporal resolution sufficient to determine changes in the environment around plaques.

In summary we could show that changes in glutamate dynamics in the early stages of AD are part of a dysfunctional microenvironment that is directly linked to plaque deposition. Even though we cannot conclude that higher levels of glutamate precede hyperactive states in different cell types it certainly contributes to the overall pathomechanism and excitotoxic effects described in the microenvironment of amyloid deposits. It could thus be used as a novel biomarker to indicate cellular dysfunction aiming at early intervention that has the potential to stop or delay detrimental downstream effects caused by excitotoxicity.

## Methods

### Mice

Hemizygous APPPS1 mice^42^ that express human APPKM670/671NL and PS1L166P under the control of the Thy-1 promoter were injected with 1 μl AAV1.hSyn.iGluSnFR.WPRE.SV40 in either somatosensory cortex (hindlimb region) or primary visual cortex. The line was generated on a C57BL/6 background. For this study, APPPS1 (±) and age-matched WT animals (C57BL/6J, 5–6 months), male and female mice, were used (APPPS1 *n*=7–9, WT *n*=7–9, 3–7 locations within the respective brain areas were acquired per mouse). Mice were group housed under pathogen-free conditions. After surgery, mice were singly housed. All procedures were conducted in accordance with the guidelines from the Canadian Council for Animal Care, and were conducted with approval from the University of British Columbia Animal Care Committee.

### Surgery

An intracortical injection of 1 μl AAV1.hSynapsin.iGluSnFR (virus titre 2.56e13 GC per ml) was performed under general anaesthesia (1–1.5% isoflurane) using a stereotactic frame (Stoelting, Model 51731) and a microinjection system (Drummond Nanoject). Two different brain areas were targeted. Mice (*n*=9 per group) were injected in the hindlimb region of the somatosensory cortex (bregma −0.6, −1.5 lateral of midline, depth 400 μm) and a second cohort (*n*=7 per group) was injected in primary visual cortex (bregma −2.8, lateral from midline −2.3, depth 400 μm). For simultaneous expression of iGluSnFR and jRGECO a mixture of 0.5 μl AAV1.hSynapsin.iGluSnFR and 0.5 μl of AAV1.Syn.Flex.NES-jRGECO1a.WPRE.SV40 (ref. 47) was injected into the hindlimb region of the somatosensory cortex (bregma −0.6, −1.5 lateral of midline, depth 400 μm, APPPS1 mice *n*=3, 3–4 imaged brain areas per mouse). After 10 days of recovery, a round cranial window (4 mm diameter) was installed under general anaesthesia (fentanyl, 0.05 mg kg^−1^; midazolam, 5 mg kg^−1^; and medetomidine, 0.50 mg kg^−1^) as described previously^14^,^41^. Imaging began 1 week post surgery. Before experiments in the awake state, animals were acclimated to head restraint in the imaging system for a minimum of 1 week after recovering from surgery. As previously reported, we ensured that neither microglia nor astrocytes were activated due to inflammation at this timepoint after the surgery^14^,^41^,^48^.

### Ceftriaxone and vehicle treatment

An intraperitoneal injection of 200 mg kg^−1^ of ceftriaxone was given for 5 consecutive days to APPPS1 and WT animals (*n*=7–9 per group)^32^,^34^,^35^. Ceftriaxone was dissolved in saline containing 1% dimethylsulphoxide. Control groups of WT and APPPS1 animals (*n*=5 per group) were given an intraperitoneal injection of vehicle for 5 consecutive days.

### Imaging

*In vivo* imaging was performed on awake and anaesthetized mice (1.0% isoflurane) using a custom-built, fully motorized, two-photon microscope^49^ equipped with a Coherent Chameleon Ultra II laser and a Zeiss W Plan-Apochromat × 40/numerical aperture 1.0 objective. The mice were secured under the microscope by fitting the titanium ring in a custom-built head fixation apparatus, which was specifically designed for high repeatability allowing fields of view to be relocated using saved coordinates^41^. The scope is motorized and controlled by a Sutter MP285 via ScanImage (version 3.8)^49^. Methoxy_X04 and iGluSnFR were imaged separately using 800 and 920 nm excitation wavelength and were detected via non-descanned detectors and ET525/50m-2P and ET605/70m-2P emission filters (Chroma Technology). iGluSnFR images were collected using ScanImage at 128 × 128 pixels without averaging at a depth of 100–140 μm below the cortical surface. Linescan (16 × 128 pixels) tplsm was performed at 57 Hz. A total of 3–7 fields were imaged per mouse. Laser power was kept constant for each experiment and did not exceed 45 mW. All shown iGluSnFR images were taken at a relatively high scan speed of 5.92 Hz with only a 128 × 128 resolution. We thus willingly decreased the resolution of the image to gain scanning speed, which was necessary to track changes in relative glutamate fluorescence on sensory stimulation. Our tplsm was always performed at the same depth and thus only detected signals generated in layer 2/3. To image the area of maximum evoked responses using tplsm all animals were mapped with wide-field imaging before tplsm data acquisition. The area of the highest response was marked by using surface vasculature, which was subsequently easily identifiable using a low-magnification objective on the tplsm. This way we ensured that the responses that were detected with tplsm came from the primary response area seen in wide-field imaging.

### Wide-field imaging

After a period of recovery post-cranial window surgery animals were anaesthetized with isoflurane (1%). Images (12-bit with 6.67 ms temporal resolution) were captured on a charge-coupled device camera (1M60 Pantera, Dalsa). A hindlimb stimulus was given mechanically by the use of a piezo device (single tap, 1 ms). For each stimulus five trials were averaged^50^. The wide-field method combines fluorescence originating from all layers of the cortex due to the large depth of field of the macro lens^50^. In contrast to two-photon imaging of layer 2/3, charge-coupled device -based wide-field imaging obtained signals within a large depth of focus spanning more cortical layers and a larger volume of tissue.

### Sensory stimulation

*Somatosensory cortex*. Animals were head-fixed and anaesthetized with isoflurane (∼1%) during imaging. A hindlimb stimulus was given mechanically by the use of a piezo device (1 ms, single tap).

*Visual cortex*. Mice were head-fixed awake and were allowed to run on a wheel where they received a visual stimulus in the form of a blue LED light flash (100 ms pulse) to the left eye. Before two-photon imaging the area of the largest visual response was mapped using wide-field imaging described above.

### Analysis

Imaged iGluSnFR fluorescence intensity was used to calculate responses to stimulation as the fractional change in fluorescence intensity and reported as % Δ*F*/*F*_0_. For both hindlimb and visual stimulation 10 trials were collected and averaged. To quantify the magnitude of signal fluctuation at each pixel over time we calculated the r.m.s. of the iGluSnFR Δ*F*/*F*_0_ using ImageJ. The r.m.s. maps are displayed as false colour heat maps with warm colours representing relatively higher fluctuations in the iGluSnFR signal at that pixel and colder colours representing smaller iGluSnFR fluctuations. Four regions of interest (20 × 35 μm) were analysed in each field of view. The ROIs were positioned systematically using the following work flow:

1. Find the largest response within the periphery of a plaque 80 μm away from the edge of the plaque. Place a 20 × 35 μm ROI spanning from 80–100 μm distance to the plaque edge. This is the position of ROI4.
2. Place putative ROIs 1–3 along the line connecting the centre of ROI4 and the edge of the plaque.
3. If any of ROIs 1–3 showed no response >1 s.d. above the baseline these ROIs were moved azimuthally around the plaque at the appropriate distance.
4. If a response is located update the ROI position.
5. Continue to update the ROI location if a larger response is found.

If no response is located in 360°, leave the ROI at the original location.

Analysis in WT animals was performed using one ROI of the same dimensions (20 × 35 μm). Custom-written Matlab (Mathworks) programmes were used to quantify properties of the time courses at each ROI. The maximum response was located in the seven frames recorded immediately post stimulus. The noise floor of the custom-built two-photon set-up was measured to quantify the contribution of instrumentation noise to the r.m.s. maps. These noise sources account for only 0.01% Δ*F*/*F*_0_ r.m.s. To measure the baseline fluorescence, the first 5–20 frames of the trials were averaged in ROIs 1 and 4. The reported values are the mean of all animals.

Decay constants were fit using the MATLAB fit function after averaging across plaques (to reduce noise and increase the reliability of the fit) and normalizing to the response in ROI4. Values reported for tau are the best fit and its standard error using the nonlinear least-squared method. These are quantities represented by bar graphs and their error bars in the figures.

### Statistical analysis

A one-way analysis of variance with *post hoc* Tukey test was used for statistical analysis of maximum response, r.m.s. of Δ*F*/*F*_0_ and area under the curve. Animals that were imaged before and after ceftriaxone treatment were considered paired groups in a two-way analysis of variance with *post hoc* Tukey test. All statistical calculations were done in GraphPad Prism software. All *P* values ≤0.05 were considered statistically significant. Data are expressed as the mean±s.e.m.

### Histology

Mice were perfused intracardially with 0.1 M phosphate-buffered saline (PBS) and 4% paraformaldehyde. Brains were post-fixed in paraformaldehyde and transferred to 30% sucrose in PBS, then cryosectioned (40 μm) as free-floating coronal slices. Sections were incubated with blocking solution (10% normal goat serum and 0.4% Triton X in PBS), followed by primary antibody against EAAT2 (1 μg ml^−1^, Santa Cruz, Dilution 1:200) or GFAP (2 μg ml^−1^, Life Technologies, Dilution 1:250) and secondary antibody (Alexa-546-conjugated anti-rabbit or anti-rat IgG, 4 mg ml^−1^, Molecular Probes, Dilution 1:500). Plaques were stained with methoxy-X04. Sections were imaged using an Olympus FV1000 confocal microscope and quantification was carried out using custom Matlab scripts. Mature and prefibrillar amyloid was stained by hFTAA (3 μM, hepta-formylthiophene acetic acid).

### Data availability

The data that support the findings of this study are available from the corresponding author on request.

## Additional information

**How to cite this article:** Hefendehl, J. K. *et al*. Mapping synaptic glutamate transporter dysfunction *in vivo* to regions surrounding Aβ plaques by iGluSnFR two-photon imaging. *Nat. Commun.* **7,** 13441 doi: 10.1038/ncomms13441 (2016).

## Supplementary Material

Supplementary Information Supplementary Figures 1-7

Supplementary movie 1 Wide field imaging movie shows the prestimulus baseline followed by the increase in iGluSnFR fluorescence upon hindlimb stimulation and subsequent recovery to baseline levels in false colours.
