# Supplementary material for: Mapping synaptic glutamate transporter dysfunction in vivo to regions surrounding Aβ plaques by iGluSnFR two-photon imaging
Source: Nat Commun. 2016 Nov 11;7:13441. doi: 10.1038/ncomms13441 (PMC5114608; doi:10.1038/ncomms13441)
Supplement: Supplementary Information — Supplementary Figures 1-7 [file ncomms13441-s1.pdf]

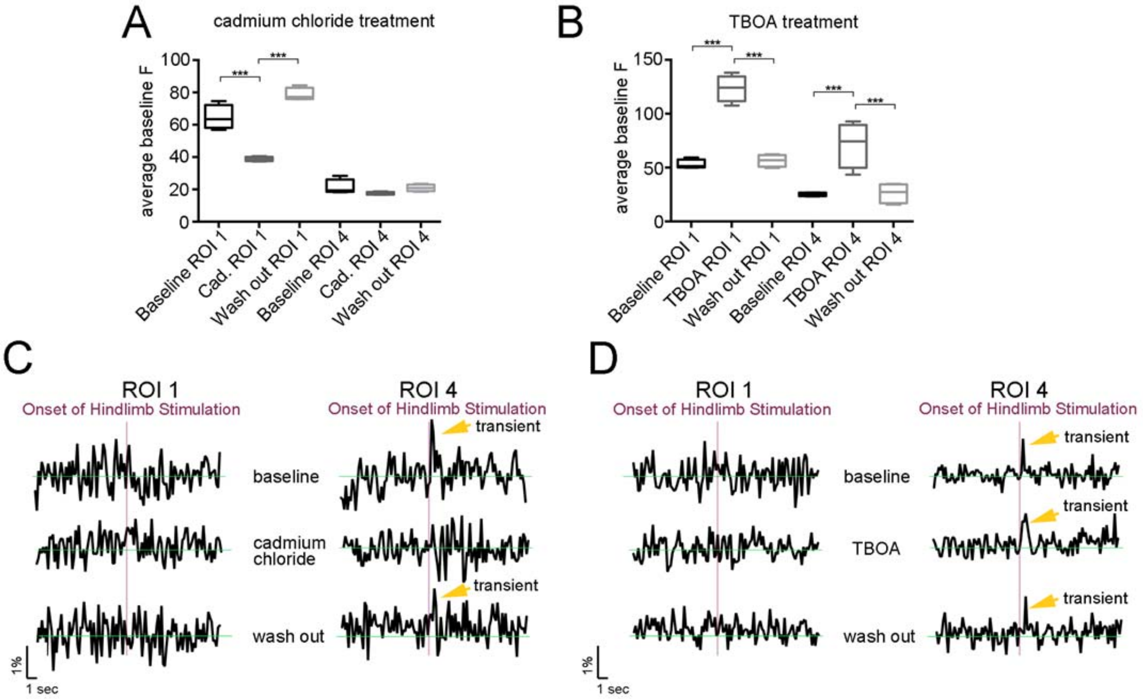

Supplementary Fig.1 Measurement of iGluSnFR signal fluorescence in plaque microenvironment and manipulation of glutamate signaling pathway

Intracortical injection of AAV.hSynapsin.iGluSnFR in somatosensory cortex area (APPPS1 n=7-9, 3-7 imaged brain areas/mouse). A) Fluorescence of iGluSnFR to calculate relative glutamate levels was measured in ROI1 and ROI4 by averaging baseline frames 1-25 over all APPPS1 animals. ROI1 shows a significantly higher level of fluorescence in comparison to ROI4 pointing towards higher glutamate levels within this area. B-E) Open brain experiments after intracortical injection of AAV.hSynapsin.iGluSnFR in somatosensory cortex area (APPPS1 n=2, 2 imaged brain areas/mouse) were used to manipulate the glutamate pathway. B) Cadmium chloride (Cd^{2+}, 1mM) was applied topically to the brain (dura mater was left intact) to block all presynaptic transmitter release and thus release of glutamate. The detected levels of glutamate fluorescence changed upon treatment. Levels in ROI1 were significantly reduced however stayed elevated in comparison to ROI4 levels. C) Treatment with TBOA was used to block glutamate uptake. In this experiment fluorescent levels of ROI1 and ROI4 were significantly increased, with ROI1 displaying permanently higher levels throughout the baseline, treatment and wash-out phase. Both treatments could be reversed after a wash-out phase of 10 min. D-E) Confirmation of the change in glutamate signaling can be seen in the average traces of all recorded animals. Upon application of Cd^{2+} the sensory evoked response is abolished and returns after a 10 min wash-out phase (panel D). Upon treatment with TBOA the hindlimb response is longer and wider resulting in a longer rate of decay which also reversed to its baseline level after a 10 min wash- out phase. ***P<0.001, **P<0.01, *P<0.05.

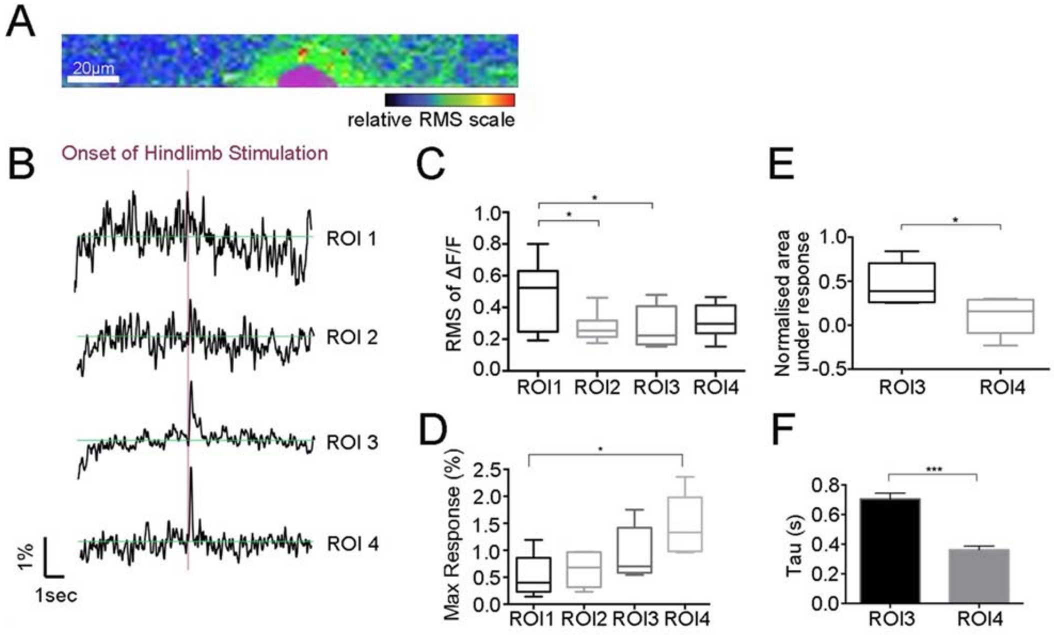

Supplementary Fig.2 Detection of altered glutamate dynamics in Aβ plaque microenvironment using tplsm linescan imaging

A) Intracortical injection of AAV.hSynapsin.iGluSnFR in somatosensory cortex area (APPPS1 mice n=9, 3-4 imaged brain areas/mouse). Glutamate fluctuations were imaged using linescan tplsm (16x128 Pixels, 57Hz). Relative overall fluctuation of glutamate is shown in false colour RMS map. Methoxy_X04 stained amyloid deposit is coloured in purple. APPPS1 animals show altered glutamatergic activity near plaque. Scalebar 20µm B) Traces representing glutamate dynamics differ significantly in relation to Aβ plaque distance. High fluctuations were measured in ROI1 in which no stimulus locked response was detected. ROI3 displayed a stimulus-evoked response with significantly altered characteristics in comparison to ROI4. C) The highest average RMS was measured in the direct vicinity of Aβ plaques with its average RMS of ΔF/F being significantly different in comparison to all other ROIs. D) The maximal response to the stimulation was detected in the ROI imaged farthest away from the Aβ plaque (ROI4). E) A significant difference in the area under the response between ROI3 and ROI4 was detected. F) The decay rates of glutamate calculated for ROIs3 and 4 are significantly different. Error bars indicate SEM. ***P<0.001, **P<0.01, *P<0.05.

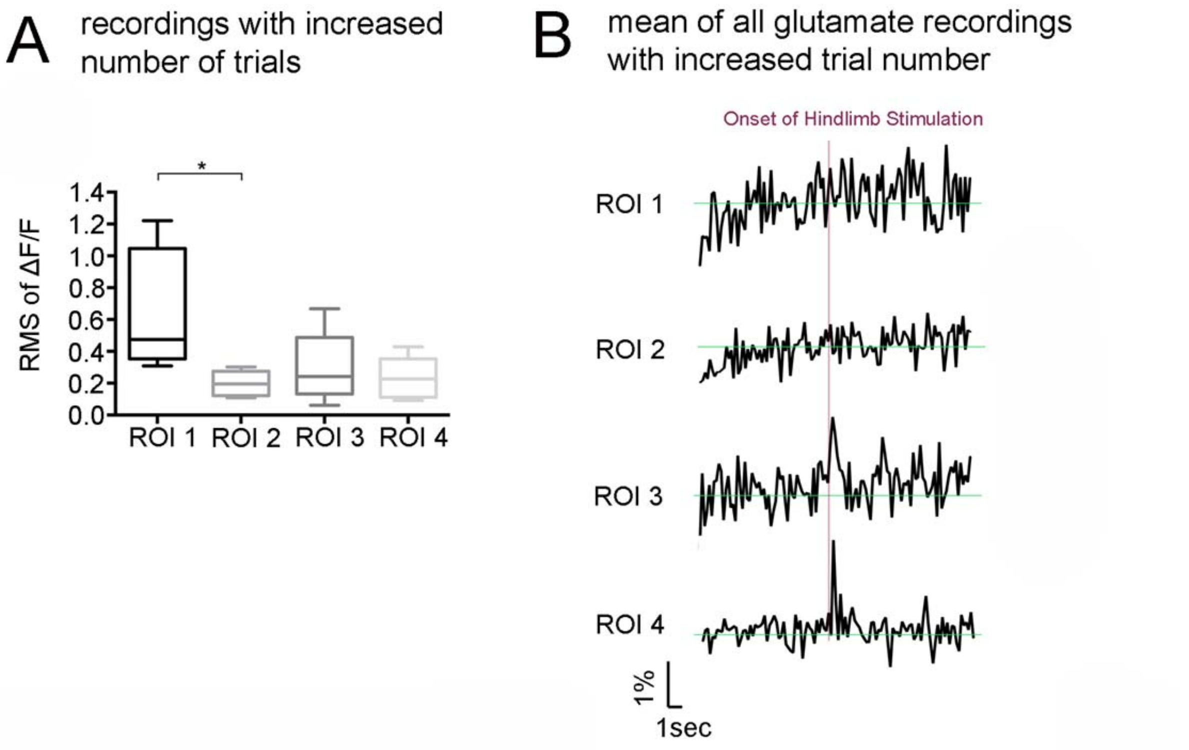

Supplementary Fig.3 Reduction of possible background noise in ROI1

A) To exclude the possibility that a stimulus evoked response of glutamate is hidden in the high fluctuation in ROI1, the hindlimb stimulation was repeated using 100 trials. RMS of average RMS of ΔF/F was still significantly higher in ROI1 in comparison to all other ROIS and the mean of all measured glutamate traces did still not result in a stimulus locked response in ROI1 and 2 B). ***P<0.001, **P<0.01, *P<0.05.

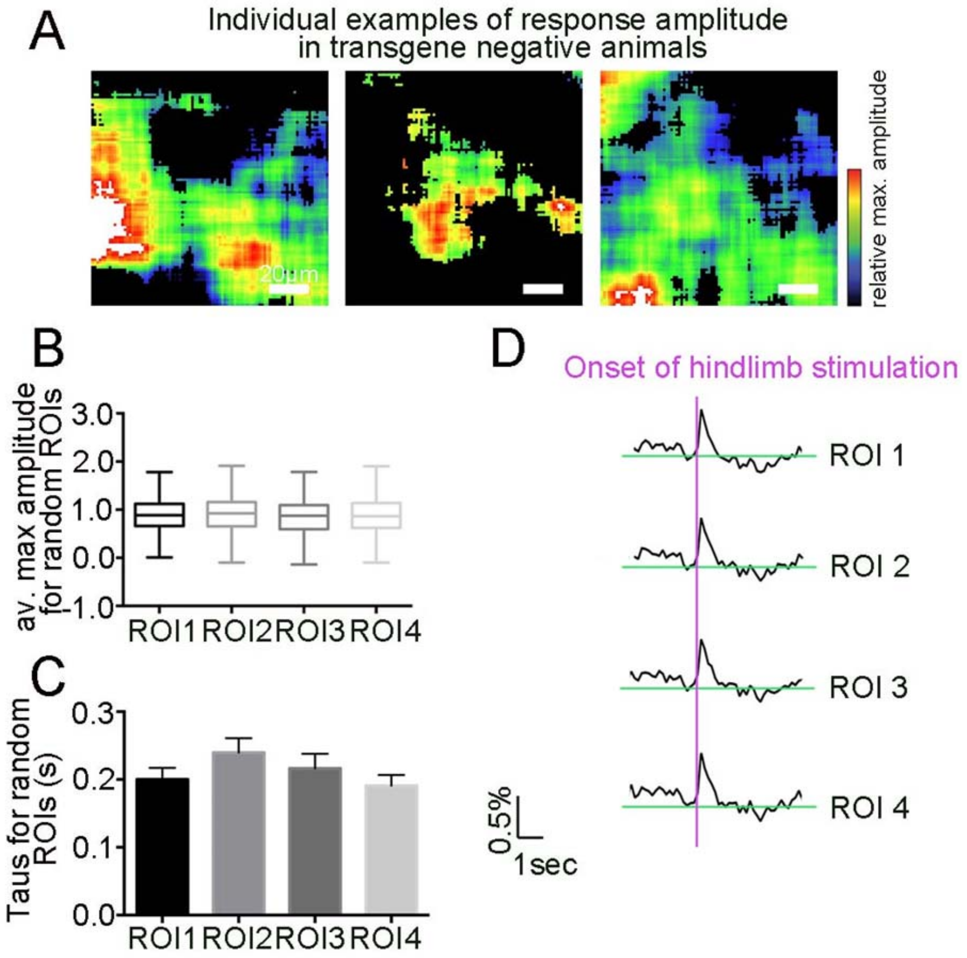

Supplementary Fig.4 Non-uniform response to hindlimb stimulation in transgene negative animals

A) Three individual examples of transgene negative animals responding to a hindlimb stimulus. The responding areas are shown in false colour displaying the relative maximum amplitudes at the timepoint of stimulation. B) The randomly placed ROIs show an average maximum amplitude that is not significantly different when comparing the ROIs. C) The taus obtained from the randomly placed ROIs show no significant difference when comparing the different traces, as can also be seen in the individually plotted average traces in D. Error bars indicate SEM.

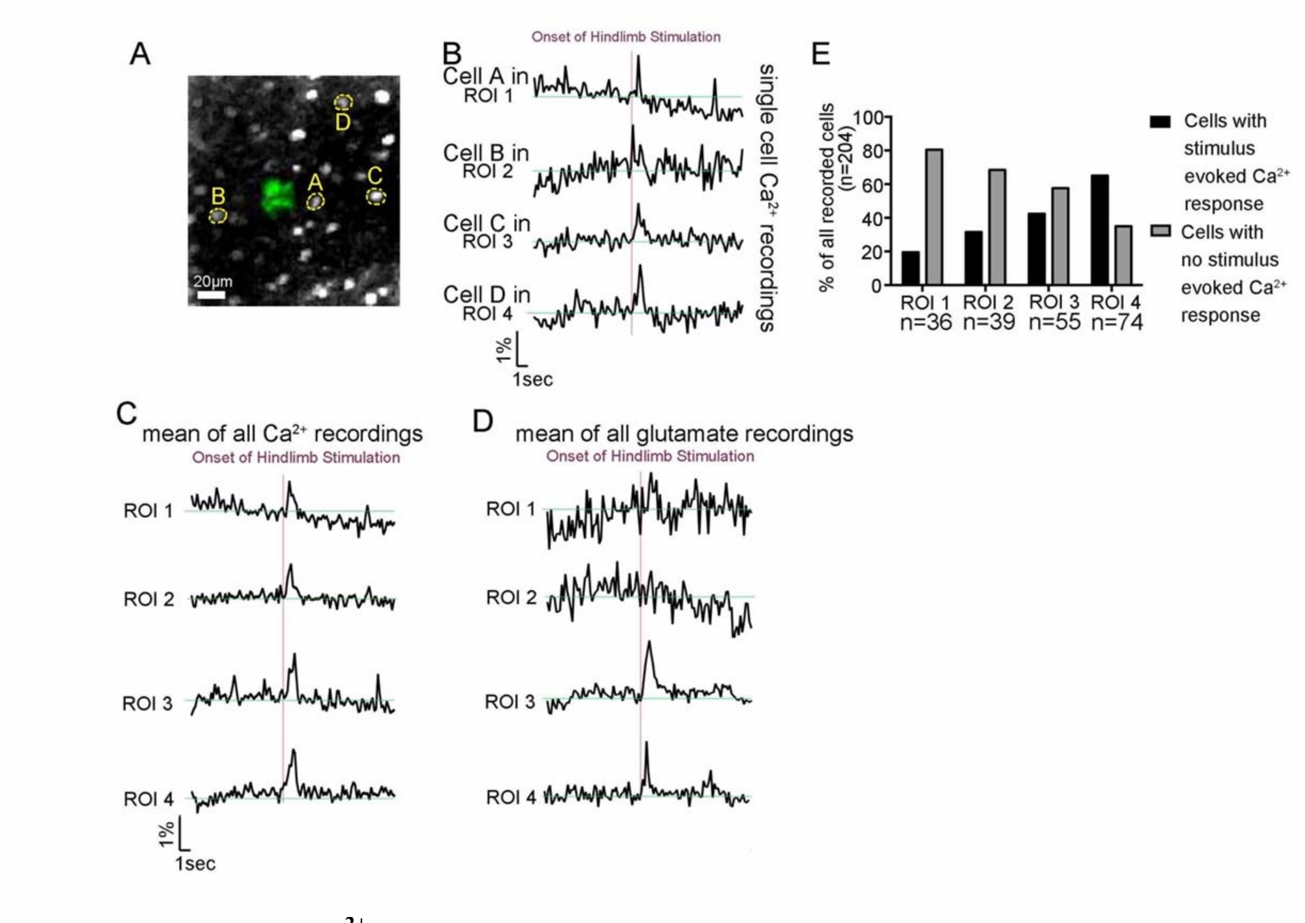

Supplementary Fig. 5 Ca^{2+} imaging in relation to plaque distance

Intracortical injection of AAV.hSynapsin.iGluSnFR and AAV1.Syn.Flex.NES-jRGECO1a.WPRE.SV40 in somatosensory cortex area (APPPS1 mice n=3, 3-4 imaged brain areas/mouse). Glutamate and Calcium fluctuations were imaged using tplsm. A) Merge of brain region with jRGECO expressing neurons (white) and methoxy_X04 stained amyloid deposit (green). Scalebar 20µm. B) Traces of single calcium recordings (circled in yellow) in different regions of interest in relation to the amyloid deposit shown in green. C) Mean of recorded Ca^{2+} traces from all responding cells in the different regions of interest. D) Mean of recorded Glutamate traces from the different regions of interest. No stimulus evoked response was detected in ROI1 and 2. E) Even though Ca^{2+} recordings showed stimulus evoked responses in all ROIs, the overall percentage of responding cells was lower in the immediate plaque microenvironment.

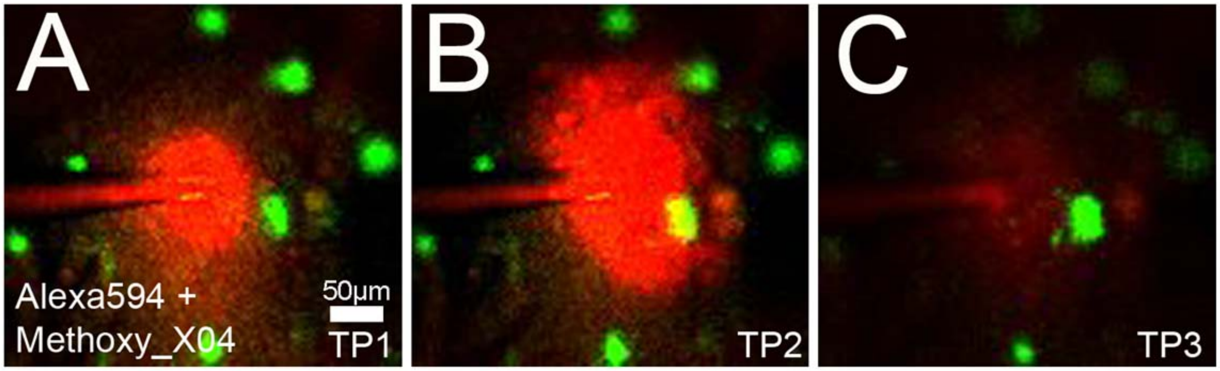

Supplementary Fig.6 Diffusion of Alexa594 around amyloid plaques

Alexa 594 was puffed into the brain adjacent to an amyloid plaque to test whether the rate of diffusion and or rate of decay is changed in ROI1 in comparison to ROI4. A) Alexa 594 is shown in red and amyloid plaques are stained with methoxy_X04 in green. Time point 1 (TP1) shows the start of the diffusion. B) The Alexa 594 is reaching the amyloid plaque in TP2. C) The decay of the dye around the plaque is shown in TP3. The following taus were calculated for the decay rate of Alexa 594 in ROI1 tau=11.290+/-0.2 sec and ROI4 tau=11.30+/-0.2 sec. Thus, no difference in the rate of decay was detected in this experiment.

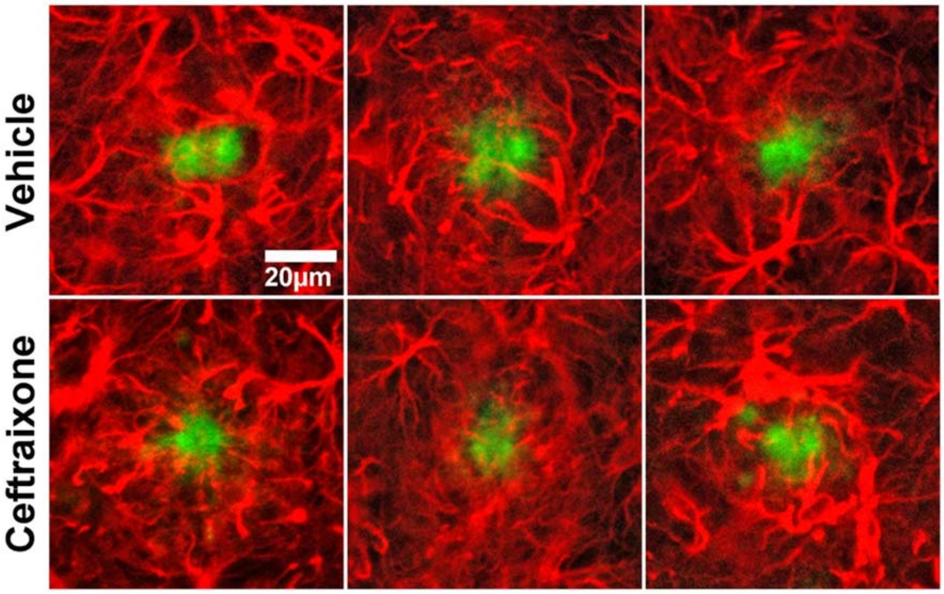

Supplementary Fig.7 GFAP staining around amyloid deposits after Ceftriaxone and vehicle treatment

Immunohistological stainings of astrocytes using GFAP in red and amyloid deposits in green are shown in 3 different examples after treatment with either Ceftriaxone or vehicle.
We observed a similar expression pattern when comparing GFAP after Ceftriaxone and vehicle treatment.
